# Neural Networks of Unconscious Processes: A Systematic Review of Functional Connectivity in Dreams and Free Association

**DOI:** 10.7759/cureus.102771

**Published:** 2026-02-01

**Authors:** Héctor Aceituno, Juan José Valero, Luisana Maldonado-De Santiago, René Mena-González, Andrés Rojas, José Villamediana-Rodríguez, Francisco Rico-Fernández, Juan Lopéz-Urdaneta, Anthuan Hazkour

**Affiliations:** 1 Neurosurgery, Hospital San Juan de Dios de Curicó, Curicó, CHL; 2 Neurosurgery, Instituto Clínico La Florida, Caracas, VEN; 3 Psychiatry, Hospital San Juan de Dios de Curicó, Curicó, CHL

**Keywords:** default mode network, functional neuroimaging, neural connectivity, neuropsychoanalysis, unconscious processes

## Abstract

Although previous neurophysiological studies validated aspects of unconscious processing, a detailed characterization of specific neural circuits required modern functional neuroimaging techniques. This systematic review examines the shared neural substrates between dreaming and free association of ideas, two paradigmatic manifestations of unconscious processing. A systematic search was conducted in PubMed, Scopus, and Web of Science (last 25 years) using terms related to functional neuroimaging, REM (rapid eye movement) sleep/NREM (non‑rapid eye movement) sleep, and free association. A total of 28 studies meeting specific inclusion criteria were included: use of advanced neuroimaging techniques (functional magnetic resonance imaging (fMRI), electroencephalography (EEG), intracranial electroencephalography (iEEG), and positron emission tomography (PET)), research in humans on unconscious processes, and explicit linkage to functional neuroanatomy. Bias assessment was performed using the Newcastle-Ottawa scale. Both processes converge in the activation of the default mode network (DMN), specifically the medial prefrontal cortex, posterior cingulate cortex, and precuneus. During REM sleep, hyperactivation of the amygdalo-hippocampal complex is observed with functional disconnection of the dorsolateral prefrontal cortex (DLPFC). Free association shows similar patterns: activation of the anterior temporal lobe and angular gyrus with reduced prefrontal modulation. Functional microstates in REM reveal oscillations between tonic (residual sensory processing) and phasic (environmental isolation) periods. Thalamo-cortical connectivity modulates narrative content generation in both states. The findings empirically validate psychoanalytic concepts through the identification of shared neural circuits. Prefrontal deactivation facilitates the expression of associations embedded in deep semantic networks, released from executive control. These results establish neurobiological foundations for contemporary neuropsychoanalysis and suggest specific therapeutic targets for interventions in unconscious processing disorders.

## Introduction and background

Sigmund Freud's The Interpretation of Dreams, published in 1900, forever changed our understanding of the mind. This seminal work revolutionized the idea of the unconscious, transforming it into something that could be observed and analyzed scientifically, beyond a mere philosophical construct. Freud showed us that even in a state of rest, mental activity continues, which led to a revolution in the understanding of psychic processes [1-3].

The Unconscious is the most extensive psychic system, containing repressed desires and impulses incompatible with reality that determine neurotic symptoms and parapraxes. The Preconscious functions as an intermediate zone with latent contents accessible through conscious attention. The Conscious represents the superficial layer limited to current perceptions and thoughts of the attentional focus. Defense mechanisms prevent direct access to the Unconscious, which is why Freud developed free association, an uncensored verbalization method in which individuals articulate thoughts exactly as they arise, without filtering or logical structuring, as a technique to overcome resistances [1-3]. This topographic structure laid the foundation for the psychoanalytic understanding of repression and clinical interpretive practice [1-3].

His research was fundamental in demonstrating that dreams are a direct expression of the unconscious mind, acting as a bridge between forms of consciousness. His theory emphasized the famous mechanisms of condensation and displacement, the ways in which conscious reality is transformed and expressed as dream content, where condensation refers to a primary process through which multiple latent thoughts, wishes, or memories are compressed into a single manifest element, making dream imagery dense, symbolic, or overdetermined, and displacement denotes the shifting of emotional intensity from a significant or threatening idea to a safer or trivial one, producing dreams in which minor details carry strong affect while central conflicts appear minimized or absent [1-3].

In 1971, Spanish psychologist Luis Cencillo challenged Freud's conceptualization of the unconscious as primarily a repository of repressed material, proposing instead "unconscious life" as an active system of affective-cognitive processing where affects and sensations predominate over logical thinking [4,5]. Cencillo described personalized "codes for interpreting reality," idiosyncratic patterns operating largely outside awareness that shape individual experience [4-6]. Contemporary neuroimaging findings, showing coordinated activity within the default mode network (DMN), dynamic interactions between limbic and associative cortices, and reduced executive control during spontaneous thought, invite comparison with earlier psychological theories emphasizing dynamic unconscious processes [7,8].

Neuroanatomical research has identified several structures that contribute to unconscious processing, forming a distributed system that shapes emotion, memory, and behavior outside of awareness. The amygdala supports rapid, implicit evaluation of threat and emotional salience, while the basal ganglia mediate habitual responding and procedural learning that unfold automatically. Parietal regions contribute to pre‑attentive sensory integration and spatial representations that guide action before conscious perception. In contrast, the prefrontal cortex, particularly its ventromedial and orbitofrontal subdivisions, integrates implicit reward and valuation signals, whereas reduced dorsolateral prefrontal activity is associated with diminished executive oversight during spontaneous or unconscious mentation. The hippocampus contributes contextual and associative memory fragments that can emerge without deliberate recall, and the broader limbic system coordinates affective and motivational processes that influence cognition beneath awareness. Together, these interconnected regions provide the neurobiological substrate for dynamic unconscious processes relevant to dreaming, free association, and spontaneous thought [7-9].

Several aspects of his framework show interesting parallels with modern observations, though these emerged from clinical intuition rather than neuroscientific prediction. His emphasis on active unconscious processing resonates with findings of persistent DMN activity during rest and sleep: a large‑scale intrinsic network implicated in self‑referential processing, autobiographical memory retrieval, and the generation of spontaneous internally directed thought [7,8]. His description of affective primacy finds potential correlates in amygdalo-hippocampal hyperactivation during REM (rapid eye movement) sleep [9]. His concept of personalized frameworks anticipates recognition of substantial inter-individual variability in DMN connectivity patterns [7].

However, these apparent convergences require careful interpretation. Cencillo's concepts operated at the phenomenological level, while neuroimaging reflects hemodynamic correlates, fundamentally different levels of analysis [4,5]. The correspondence might reflect successful clinical intuitions about mental architecture, the flexibility of psychoanalytic concepts allowing post-hoc mapping onto diverse findings, or genuine but partial overlap. Distinguishing these possibilities requires experimental paradigms specifically designed to operationalize psychoanalytic predictions in neurobiological terms, work largely absent from current literature. Both traditions recognize substantial mental activity outside conscious awareness, though they conceptualize it at different levels of analysis. In Cencillo's framework, unconscious processes are described phenomenologically as "codes for interpreting reality," idiosyncratic, pre-reflective patterns that shape experience without entering awareness [6]. Contemporary neuroimaging, by contrast, identifies large-scale networks such as the DMN, limbic circuits, and associative cortices whose spontaneous, internally generated activity unfolds independently of deliberate control, indicating robust non-conscious processing during rest, dreaming, and free association [7-9]. Although these perspectives converge in affirming that much mental life occurs outside conscious monitoring, they do so from fundamentally different epistemic standpoints: one grounded in subjective phenomenology, the other in hemodynamic and connectivity-based markers of neural activity. Clarifying how these domains relate, whether psychoanalytic constructs anticipate genuine neural dynamics, reflect flexible interpretive schemas, or capture only partial overlaps, requires experimental paradigms explicitly designed to translate psychoanalytic predictions into operational neurobiological terms, a line of research still largely undeveloped in the current literature.

Some of the most studied domains regarding the underlying structures of unconscious processes are dreaming and the free association of ideas, both sharing common properties: they generate content spontaneously, are less monitored by executive control, and show strong correlations with brain networks such as the DMN and limbic structures [8,10]. These phenomena exemplify spontaneous thought, internally generated mental activity that arises without deliberate intention and is supported by intrinsic network dynamics, and reflect broader forms of unconscious processing, in which cognitive, emotional, and mnemonic operations unfold outside awareness through the coordinated activity of limbic, associative, and default mode systems [8,10]. Together, dreaming and free association provide accessible windows into the brain's intrinsic, nonconscious mentation, illustrating how spontaneous thought emerges from neural mechanisms that operate independently of conscious control. The modern conception of the unconscious is more nuanced than early psychoanalytic accounts, as it is largely based on neuroscientific evidence and has allowed mapping of the neural substrates of processes that used to be described primarily through clinical observations [11].

Recent advances in multimodal neuroimaging techniques and connectivity analysis have enabled the emergence of integrative models that conceptualize the unconscious mind as an emergent phenomenon arising from the dynamic interaction among multiple neural networks [12]. Thus, states of sleep and deep meditation that show significantly altered functional connectivity patterns of information compared to a quiet resting state exhibit properties somewhat similar to those of free association, with shared neural mechanisms [13].

This systematic review aims to identify and synthesize neuroimaging evidence on shared neural substrates between oneiric processes and free association of ideas, characterizing the neurobiological mechanisms of unconscious processing and establishing an integrative theoretical framework that explains the functional convergences between both cognitive phenomena. Accordingly, the central research question guiding this review is: What neural networks and functional connectivity patterns are consistently shared between dreaming and free associative thinking, and how do these overlaps inform current models of unconscious processing? Despite growing interest in spontaneous cognition, a significant research gap persists: no prior review has systematically examined whether the neural architecture underlying dreaming and free association converges in a reproducible manner across neuroimaging modalities. Existing studies remain fragmented, vary widely in methodology, and rarely address these phenomena within a unified theoretical framework, leaving unresolved how their shared features relate to broader models of unconscious mental activity.

## Review

Methodology

This systematic review, conducted in accordance with PRISMA (Preferred Reporting Items for Systematic Reviews and Meta-Analyses) guidelines, employed a comprehensive search strategy across indexed scientific databases, including PubMed, Scopus, and Web of Science. The search covered studies published between 2000 and 2025 and used the following terms combined with Boolean operators: ("dreaming" OR "REM sleep" OR "non-REM sleep") AND ("fMRI" OR "EEG" OR "PET" OR "intracranial recordings"), ("free word association" OR "semantic networks" OR "spontaneous thought") AND ("neural correlates" OR "default mode network" OR "hippocampus"), and ("predictive processing" OR "spreading activation") AND ("unconscious processing").

Inclusion Criteria

This review included peer‑reviewed empirical studies, including experimental, observational, and neuroimaging-based research, utilizing advanced techniques such as fMRI, EEG, iEEG, or PET; studies conducted in human subjects examining unconscious processes (dreams, free association of ideas); investigations reporting neuroanatomical correlates explicitly linked to unconscious processing (e.g., amygdala, basal ganglia, parietal lobe, prefrontal cortex, limbic system); articles published in English or Spanish to ensure accessibility and comprehension; and methodologically robust studies with transparent sample sizes and clearly reported statistical analyses.

Exclusion Criteria

Case reports, single-subject studies, or anecdotal evidence; animal studies unless directly translatable to human neuroanatomy; studies lacking neuroimaging or neuroanatomical data; articles with vague or incomplete methodologies; opinion section articles, editorials, or letters to the editor; duplicate publications or conference abstracts without full-text access; and studies that did not explore consciousness or irrelevant variables were excluded.

Data Extraction and Analysis

Two reviewers (H.A. and J.J.V.) independently extracted data from each included study using a standardized form specifically developed for functional neuroimaging studies. Discrepancies were resolved through discussion, and when necessary, a third reviewer (L.M.D.S.) was consulted.

Extracted data included (1) study characteristics (author, year, design, sample size), (2) participant characteristics (age, sex, inclusion/exclusion criteria), (3) neuroimaging technique employed (fMRI, EEG, PET, iEEG), (4) experimental paradigm used (REM sleep, free word association, control conditions), (5) brain regions investigated and anatomical definition method, (6) statistical analysis methods (multi-voxel pattern analysis, functional connectivity, representational similarity analysis), (7) main findings reported, and (8) methodological limitations identified by the authors.

Risk of Bias Assessment

The methodological quality of individual studies was assessed using an adapted version of the Newcastle-Ottawa Scale (NOS) for observational studies, originally developed to evaluate selection, comparability, and outcome domains in non‑randomized research. For the purposes of this review, the scale was modified to address methodological features specific to functional neuroimaging, incorporating criteria such as the adequacy of participant selection procedures, control of confounding variables, correction for multiple comparisons, the transparency and reproducibility of preprocessing pipelines, and the reporting of effect sizes. This adapted framework allowed for a more accurate appraisal of bias in studies employing fMRI, EEG, iEEG, and PET methodologies.

Results

A systematic search was conducted across multiple databases in accordance with the PRISMA 2020 guidelines. During the identification phase, a total of 34,709 records were retrieved from PubMed (n = 2,243), Web of Science (n = 80), Scopus (n = 70), PsycINFO (n = 50), arXiv (n = 16), and Google Scholar (n = 32,250) using the predefined search strategy covering publications from 2000 to 2025. Before screening, 10,065 duplicate records were removed, resulting in 24,644 unique records.

During the screening phase, 24,644 records were assessed based on their titles and abstracts. Of these, 24,397 were excluded due to irrelevance to the research question, lack of anatomical localization data, or inappropriate study models (e.g., non-human subjects, purely theoretical frameworks). This resulted in 247 reports sought for retrieval for full-text assessment.

In the eligibility phase, 156 reports could not be retrieved despite exhaustive search efforts, leaving 91 reports successfully retrieved and evaluated for eligibility. After full-text review, 53 reports were excluded due to specific exclusion criteria, including experimental design requirements, lack of human subject data, theoretical contributions without empirical evidence, and other methodological considerations.

**Figure 1:**
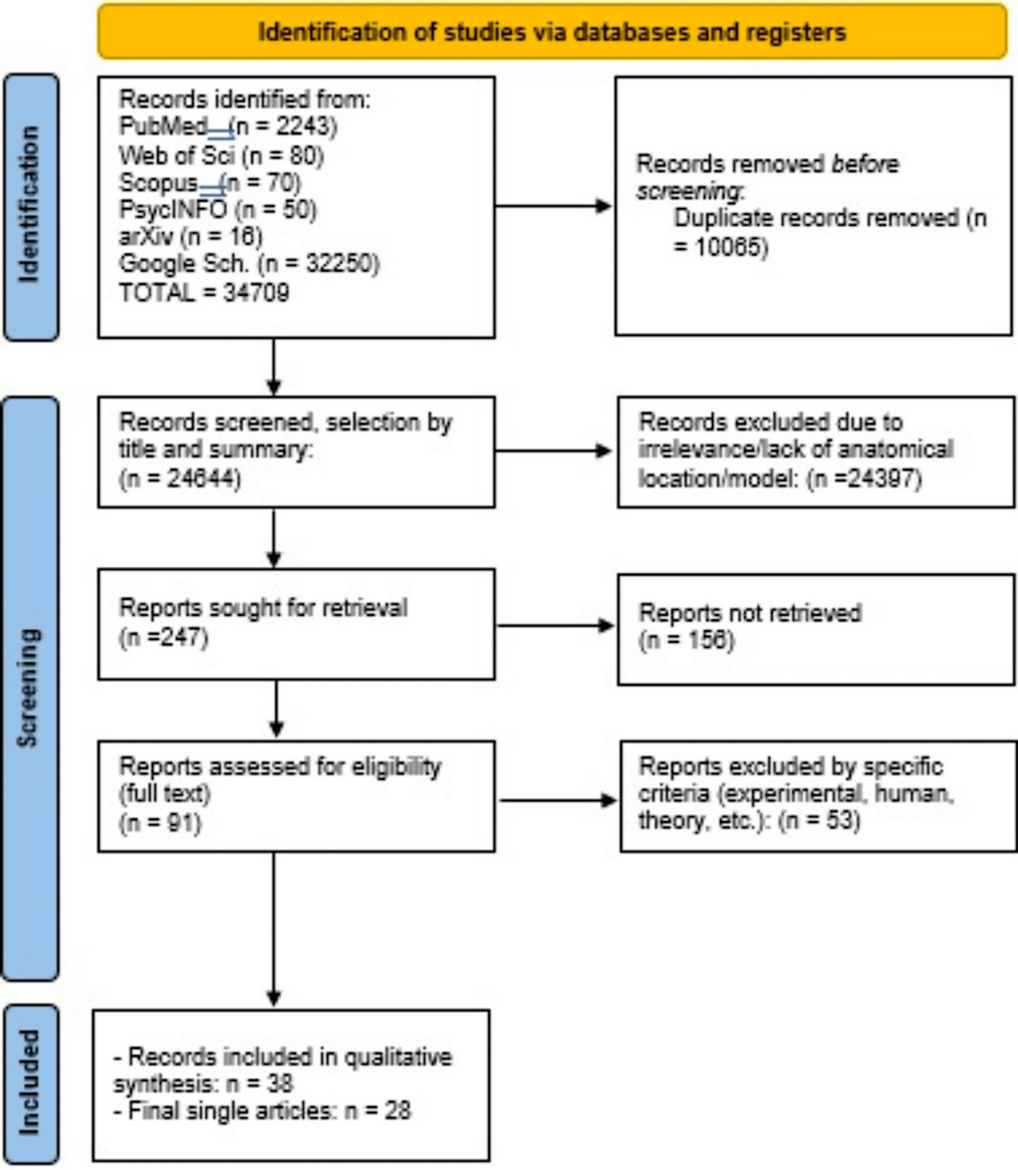
PRISMA flow diagram PRISMA: Preferred Reporting Items for Systematic Reviews and Meta-Analyses

The final inclusion phase comprised 38 studies that satisfied all eligibility criteria regarding study design, neuroimaging methodology, population characteristics, and direct relevance to unconscious processing during dreaming or free associative thinking. These 38 studies, representing 28 unique articles, were included in the qualitative synthesis to characterize shared neural substrates between oneiric processes and free associative cognition (Figure 1).

Neuroimaging studies have systematically characterized the neural architecture underlying dream generation across multiple dimensions of brain organization, revealing specific brain regions critically involved in dreaming processes. The synthesis of functional connectivity analyses, structural neuroimaging, and electrophysiological investigations reveals a hierarchical pattern of network engagement during dream processes [14-26]. Table 1 systematizes the anatomical regions, their functional contributions, characteristic activation patterns, and sleep-stage specificity documented in the reviewed literature.

**Table 1:** Neural Activation Findings in Dream Processes DMN: Default mode network, REM: Rapid eye movement, NREM: Non-rapid eye movement, mPFC: Medial prefrontal cortex, PCC: Posterior cingulate cortex, TPJ: Temporoparietal junction, IPL: Inferior parietal lobules, DLPFC: Dorsolateral prefrontal cortex, DMPFC: Dorsomedial prefrontal cortex

Region/Network	Primary Function	Activation Pattern	Sleep Stage	References
Default mode network				
mPFC	Self-referential processing, autobiographical memory	Hyperactivation	REM, NREM	[14,15,17,18,22,25]
PCC	Integration of internally-generated information	Hyperactivation	REM	[14,15,18]
Precuneus	Visual imagery, episodic memory retrieval	Hyperactivation	REM	[14,15]
TPJ	Theory of mind, narrative processing	Hyperactivation	REM	[18,26]
IPL	Attention to internal states	Hyperactivation	REM	[14,15]
Limbic system				
Hippocampus	Episodic memory integration and consolidation	Activation	REM > NREM	[19,23,26]
Amygdala (bilateral)	Emotional processing and dream vividness	Hyperactivation	REM	[9,19,23]
Left amygdala	Emotional content access	Activation (MD inversely correlated with dream length)	REM	[23]
Right amygdala	Bizarreness of dream content	Activation (MD positively correlated with bizarreness)	REM	[23]
Prefrontal regions				
DLPFC	Executive control, logical reasoning	Deactivation	REM	[9,20]
DMPFC	Autobiographical regulation	Decreased connectivity with PCC	REM	[20]
Thalamo-cortical system				
Thalamus	Sensory gating, state transitions	Phasic activation	REM (phasic/tonic)	[24]
Thalamo-cortical network	Imagery and narrative generation	Oscillatory activation	REM	[24]
Temporo-occipito-parietal				
Occipital-temporal-parietal regions	Transformation of perceptions into concepts	Activation	REM	[21]
Visual cortex	Dream imagery generation	Activation	REM	[17,24]
Lateral temporal cortex	Semantic processing, language	Activation	REM	[14,15]

The DMN constitutes the foundational architecture for spontaneous mental content generation during dreaming [14,15]. Functional neuroimaging demonstrates consistent hyperactivation of the medial prefrontal cortex, posterior cingulate cortex, precuneus, and temporoparietal junction during REM sleep; these regions exhibit synchronized activity patterns that facilitate internally focused cognition independent of external sensory input [16-18], with maintained activity during NREM dream episodes [19-22]. The limbic system, particularly the amygdala and hippocampal formation, modulates the emotional valence and mnemonic content of dreams through selective hyperactivation during REM periods [9,19,23]. Structural imaging studies reveal that microstructural integrity in these regions correlates with specific qualitative characteristics of dream content, including emotional load, narrative coherence, and bizarreness [23]. Notably, prefrontal regions demonstrate a characteristic deactivation pattern, with the dorsolateral prefrontal cortex (DLPFC) showing a systematic reduction in activity that corresponds to the diminished executive control and logical inconsistencies observed in dream phenomenology [9,20]. The thalamo-cortical system exhibits distinctive phasic and tonic activation patterns that differentiate periods of sensory isolation from residual environmental monitoring capacity [24]. Finally, the temporo-occipito-parietal regions transform abstract cognitive content into the vivid perceptual experiences characteristic of dreaming, effectively reversing the wakeful process of sensory abstraction [21].

The systematic organization of these findings in Table 1 reveals several critical patterns. First, the convergence of activation across DMN structures during both REM and NREM dreaming suggests that the neural substrate for spontaneous thought generation operates relatively independently of sleep-stage architecture [18,25]. Second, the consistent amygdalar hyperactivation coupled with hippocampal engagement indicates that emotional processing and memory integration represent core computational functions during dream generation, rather than epiphenomenal features [19,23,26]. Third, the systematic prefrontal deactivation, particularly in dorsolateral regions, provides a neural mechanism for the reduced metacognitive awareness and logical evaluation characteristic of the dream state [9,20]. Fourth, the lateralization patterns observed in amygdala function, with distinct contributions from left and right structures to emotional access versus bizarreness generation, suggest hemispheric specialization in dream content modulation [23]. Finally, the identification of thalamo-cortical microstates differentiating tonic and phasic REM periods provides a temporal framework for understanding the oscillatory nature of dream intensity and sensory incorporation [24].

Differences in connectivity between wakefulness, NREM sleep, and REM states are described, with emphasis on the connection between the medial prefrontal cortex and limbic structures (hippocampus and amygdala) [26]. Dreams during REM sleep showed significantly more socially focused content compared to thoughts during wakefulness. This suggests that dreams may serve as a space to simulate and process social interactions, possibly contributing to the development of social cognition and "theory of mind," which refers to the capacity to attribute mental states, such as beliefs, desires, intentions, and emotions, to oneself and others, and to understand that other people have mental states that may differ from one's own [26]. Dream content during REM sleep showed more positive emotions than thoughts during wakefulness. This could be related to theories proposing a role for REM sleep in emotional processing [26]. Contrary to what might be expected, no significant differences were found between dreams and waking thoughts regarding temporal focus (past, present, future), suggesting that dreams, like thoughts during wakefulness, may involve autobiographical episodes from the past, current concerns, and future plans [26].

The neural substrates of free association reveal distinct brain regions critical to this fundamental psychoanalytic technique. Free association represents a fundamental investigative technique in psychoanalytic practice, originally developed by Freud as the "fundamental rule" of psychoanalysis, wherein patients verbalize thoughts without conscious censorship or logical organization [1].

Functional neuroimaging approaches to free association typically employ contrasts between unconstrained verbal production and structured semantic retrieval tasks, enabling isolation of neural correlates specific to spontaneous associative processes [27-31]. These investigations reveal systematic differences in regional activation and network connectivity that distinguish free association from executive-controlled language production [27-31]. Table 2 systematizes the neural regions implicated in free associative thought, their functional contributions, characteristic activation patterns relative to control conditions, and supporting empirical evidence from the reviewed literature.

**Table 2:** Neural Activation Findings in Free Association Processes ATL: Anterior temporal lobe, AG: Angular gyrus, IPFC: Inferior prefrontal cortex, DLPFC: Dorsolateral prefrontal cortex

Region/Network	Primary Function	Activation Pattern	Control Condition	References
Semantic integration hubs				
ATL	Semantic integration, conceptual knowledge	Activation	Spontaneous vs. directed speech	[27,28]
AG	Associative propagation of meanings	Activation	Free vs. structured tasks	[27,28]
Medial temporal system				
Hippocampus	Integration of episodic memories into new semantic relationships	Activation under low executive control	Free vs. constrained association	[27-29]
Medial temporal cortex	Episodic-semantic memory binding	Activation	Spontaneous association	[27-29]
Executive control network				
IPFC	Semantic memory retrieval (task-directed)	Activation in structured tasks, reduced in free association	Structured vs. free tasks	[29,30]
DLPFC	Executive control modulation	Variable; reduced in unconstrained association	Task-dependent modulation	[28,29]
Default mode network				
Medial prefrontal regions	Self-referential processing, spontaneous thought	Activation	Free association vs. external focus	[31]
Posterior medial cortex	Internal mentation	Activation	Spontaneous association	[31]
Integration networks				
DMN-Executive Network interaction	Integration of spontaneous thoughts into coherent discourse	Dynamic coupling	Free association task	[31]
Prefrontal-hippocampal connectivity	Modulation by the degree of thought spontaneity	Task-dependent variation	Spontaneity gradient	[28]
Prefrontal-semantic network coupling	Control over semantic spreading activation	Reduced coupling in free association	Low vs. high executive demand	[29,30]

The anterior temporal lobe and angular gyrus play a central role in semantic integration and associative propagation of meanings [27,28]. The hippocampus and medial temporal cortex support the integration of episodic memories into new semantic relationships, particularly under conditions of low executive control [27-29].

The neural architecture revealed in Table 2 demonstrates a systematic shift from executive-controlled to spontaneous associative processing. The anterior temporal lobe and angular gyrus emerge as critical hubs for semantic spreading activation, operating with reduced prefrontal constraint during free association [27,28]. This pattern suggests that unconstrained associative thought relies on distributed semantic networks capable of autonomous propagation independent of executive oversight. The hippocampal formation contributes episodic memory content to the associative stream, with engagement inversely proportional to prefrontal control [28,29]. Notably, the DMN coordinates spontaneous thought generation while maintaining sufficient executive network interaction to translate unconscious associations into coherent verbal output [30,31]. This dynamic balance distinguishes free association from completely unconstrained mind-wandering, preserving communicative function while minimizing executive censorship.

Beyond regional activation patterns, functional and structural connectivity analyses have elucidated the network-level organization underlying free association. These studies focus on semantic organization and the activation of linguistic networks during spontaneous associations [30,31].

The DMN plays a central role in associative thinking processes, with the DMN related to free association of ideas and its interaction with executive networks to integrate spontaneous thoughts into discourse [31]. Free association is found to involve connections between the DMN and the medial executive control network, allowing the integration of unconscious ideas with consciously accessible language production. Among these networks, the anterior temporal lobe, angular gyrus, and inferior frontal gyrus are identified as centers of associative processing [27].

The dynamic interplay between prefrontal connectivity and semantic networks reveals how spontaneity modulates neural processing. Studies have shown how connectivity between the DLPFC and hippocampus varies according to the spontaneity of verbal thought [28]. Research demonstrates that free association activates the DLPFC less compared to guided verbal fluency tasks, promoting greater hippocampal activation in more spontaneous verbal production [29,30].

The convergence of neuroimaging evidence reveals common neural mechanisms underlying both dreams and free association. The DMN emerges as a shared substrate, with both processes demonstrating self-generation of mental content without direct influence from external stimuli. The fluency, flexibility, and creativity of thought are aided by the lack of DLPFC supervision in both processes [18,19,22,32].

Predictive coding and probabilistic processing models reveal the brain processes involved in producing spontaneous mental content. Both dreams and free association utilize predictive coding mechanisms to generate coherence by predicting sensory content based on previously experienced patterns [32,33]. Semantic and episodic memory networks operate through spreading activation, accounting for the emergence of unexpected associations during both dreams and free-association tasks [27,28,31]. This mechanism is reinforced by activity in limbic and emotional networks, particularly the amygdala, which facilitates unexpected access to emotionally valenced associations in both dreams and spontaneous thought [21,23].

The reviewed neuroimaging literature reveals a complex pattern of convergence and divergence in the neural mechanisms underlying dreams and free association. Both processes engage the DMN and demonstrate reduced executive control with enhanced limbic activity, yet systematic differences emerge in the degree of prefrontal dissociation, the recruitment of semantic networks, and the integration of mnemonic content. Table 3 systematically compares these processes across multiple dimensions to clarify their shared substrates and distinctive features.

**Table 3:** Convergences and Divergences in Neural Mechanisms of Dreaming and Free Association DMN: Default mode network, mPFC: Medial prefrontal cortex, PCC: Posterior cingulate cortex, TPJ: Temporoparietal junction, DLPFC: Dorsolateral prefrontal cortex, DMPFC: Dorsomedial prefrontal cortex, REM: Rapid eye movement, ATL: Anterior temporal lobe

Aspect	Similarities	Differences	Key References
Neural Networks Architecture	Both engage DMN structures (mPFC, PCC, precuneus, TPJ); hippocampal activation for memory retrieval and integration; spontaneous content generation without external stimuli [7,8,18,22,25]	Dreams: more extensive activation of visual cortex and occipital regions [9,17]; Free Association: stronger engagement of language networks (ATL, angular gyrus, Broca's area) [27,28]	[7,-9,17,18,22,25,27,28]
Prefrontal Executive Function	Both show reduced DLPFC activity relative to goal-directed cognition, allowing spontaneous, less logically constrained content [8,17,26,28]	Dreams: consistent and pronounced DLPFC deactivation in REM sleep, enabling bizarre, illogical narratives [17,26]; Free Association: variable, context-dependent DLPFC modulation—can be voluntarily engaged [28,29,31]	[8,17,26,28,29,31]
Emotional Processing and Limbic System	Amygdala and hippocampus modulate emotional content in both processes; limbic hyperactivation relative to prefrontal control [17,23,24,26]	Dreams: more pronounced emotional intensity, especially in REM with systematic amygdala hyperactivation [23,24]; Free Association: more controlled emotional expression with preserved metacognitive awareness [31]	[17,23,24,26,31]
Memory Systems Integration	Both engage hippocampal-medial temporal lobe systems for episodic memory integration into new contexts; autobiographical elements feature prominently [13,18,25,28]	Dreams: highly fragmented, recombined memories with temporal distortions [13,18]; Free Association: more coherent memory retrieval with maintained temporal structure and semantic links [28,31]	[13,18,25,28,31]
Semantic Processing and Spreading Activation	Both utilize spreading activation through semantic networks; probabilistic associations generating unexpected connections [27,28,32]	Dreams: highly associative with extensive multisensory integration (visual, auditory, somatic) [9,18,32]; Free Association: primarily linguistic-semantic with verbal output constraints [27,28,31]	[9,18,27,28,31,32]
Consciousness and Metacognition	Both represent altered states with reduced executive control and reality monitoring compared to focused waking cognition [8,26]	Dreams: occur during sleep with minimal external awareness, often no insight into the dreaming state [9,26]; Free Association: waking state with preserved metacognitive ability to observe and reflect on the thought process [31]	[8,9,26,31]
Temporal Dynamics and Network Transitions	Both show dynamic network transitions with alternating activation patterns; functional microstates evident in both processes [10,24]	Dreams: tied to ultradian REM/NREM cycles (~90 min); distinct tonic vs. phasic periods [24,26]; Free Association: continuous process with variable spontaneity, influenced by set/setting [10,31]	[10,24,26,31]
Content Generation Mechanisms	Both generate spontaneous mental content through reduced top-down control; predictive processing frameworks apply to both [32]	Dreams: bizarre, illogical narratives violating physical/temporal constraints [18]; Free Association: more coherent discourse maintaining basic logical structure despite spontaneity [31]	[18,31,32]
Neural Connectivity Patterns	Both show DMN-limbic hyperconnectivity; reduced DMN-executive network anticorrelation [18,22,26]	Dreams: more pronounced decoupling between DLPFC and associative regions [17,26]; Free Association: maintained connectivity allowing voluntary control if needed [28,31]	[17,18,22,26,28,31]

Bias Assessment of the Selected Studies

The methodological quality and potential sources of bias in the selected studies were systematically evaluated using an adapted version of the NOS for observational studies, specifically modified to address the unique methodological challenges of functional neuroimaging research. This customized assessment framework examined critical dimensions, including adequacy of participant selection criteria, control of confounding variables, correction for multiple comparisons, transparency in preprocessing parameters, and reporting of effect sizes.

Each of the five included studies was independently evaluated against these standardized criteria to identify methodological strengths and potential limitations that could impact the validity and interpretability of neuroimaging findings. The assessment framework was designed to address both general epidemiological quality standards and neuroimaging-specific considerations, such as statistical thresholding procedures and image processing protocols.

The detailed bias assessment results are presented in Tables 4-8, with each table corresponding to one of the included studies. This comprehensive quality appraisal provides transparency regarding the methodological rigor of the neuroimaging evidence and allows readers to contextualize the strength of conclusions drawn from each study within the broader systematic review framework.

**Table 4:** Summary of Risk of Bias by Study Category PET: Positron emission tomography, fMRI: Functional magnetic resonance imaging, EEG: Electroencephalography, iEEG: Intracranial electroencephalography

Study Category	Overall Risk of Bias
Neuroimaging studies (fMRI, PET, EEG, iEEG)	Moderate
Free word association studies	Low to Moderate
Reviews and theoretical models	Moderate

**Table 5:** Neuroimaging Studies (fMRI, PET, EEG, iEEG) PET: Positron emission tomography, fMRI: Functional magnetic resonance imaging, EEG: Electroencephalography, iEEG: Intracranial electroencephalography

Strengths and Weaknesses of the Neuroimaging Studies	
Strengths	Predefined methodologies with functional connectivity analysis and brain segmentation
	Several studies with sample sizes > 50 subjects, improving statistical power [9,18-21,26,28]
	Well-established methodologies for DMN, hippocampus, amygdala, and prefrontal cortex [17,23,24,32,34]
Weaknesses	Selection bias: Small samples (N < 20) limiting statistical robustness [19,23,35]
	Selection bias: Inadequate controls to differentiate waking vs. dream states [9]
	Subjective quantification: Recall bias from self-reported dream content [19,21]
	Methodological heterogeneity: Different EEG filtering methodologies limiting comparability [24,26,36]

**Table 6:** Free Word Association Studies

Strengths and Weaknesses of Free Word Association Studies	
Strengths	Well-designed methods for semantic networks assessment [27,28]
	Adequate sample sizes (N > 30) in most cases [30,31]
Weaknesses	Process control limitations: Guided vs. spontaneous associations not differentiated [28,29]
	Cultural bias: Lexical databases biased toward specific languages [2]

**Table 7:** Reviews and Theoretical Models

Strengths and Weaknesses of Reviews and Theoretical Models	
Strengths	High-quality reviews integrating psychoanalytic and neuroscientific approaches [22,32,33,37]
	Mathematical models and computational neural networks as frameworks [19,32,37]
Weaknesses	Selection variance: Emphasis on psychoanalytic models without adequate experimental integration [38-40]
	Lack of empirical validation: Theoretical claims about prediction-error in dreams without direct experimental support [32,37]

**Table 8:** General Limitations of Current Evidence DMN: Default mode network

Limitations	
1	Limited experimental integration between dreams and free association: Few studies explicitly explore common mechanisms at the level of shared neuroanatomy, despite both constructs involving spontaneous generation.
2	Methodological challenges in dream studies: Correlating dream content with brain activity is difficult due to reliance on self-reports and challenges in isolating specific content in neuroimaging [19,21,26].
3	Absence of structural-functional correlations: While theoretical models include DMN's role in spontaneous content generation, structural-functional relationships between spontaneous and controlled processes remain unclear, leaving uncertainty about the network's responsibility for content production.

**Figure 2:**
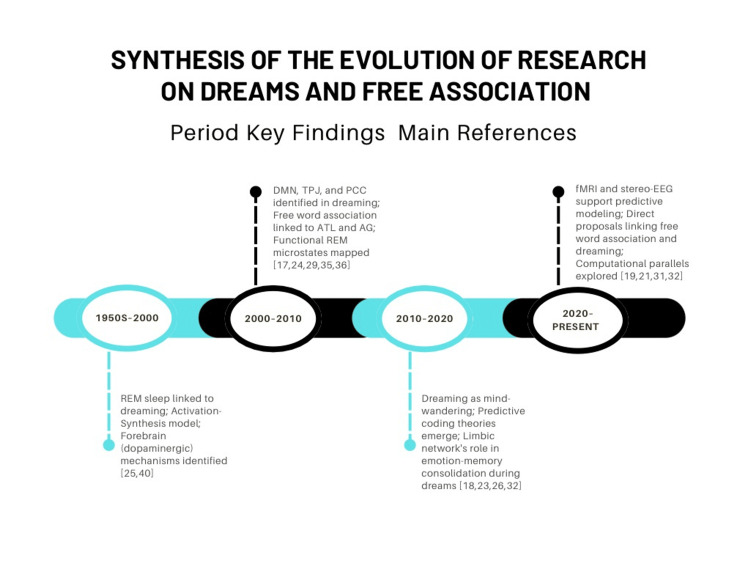
Timeline of the different techniques applied in the study of dreams and free association of ideas Image created by the authors.

Figure 2 presents a timeline of the different techniques used in the study of dreams and free association of ideas, along with their contributions to knowledge, highlighting the most relevant findings.

Discussion

Functional neuroimaging studies have characterized the neural networks underlying dream generation and free association, establishing a convergent model where the DMN, the amygdalo-hippocampal complex, and thalamo-cortical connections constitute the fundamental circuits of these unconscious processes [17,18,24,29,33]. Multimodal evidence, including intracranial recordings, functional MRI, and high-density EEG, has enabled mapping of both regional activation and dynamic connectivity patterns during these states [19,35].

Core Neural Substrates

Three fundamental components underlie unconscious processing. First, the DMN emerges as the primary architecture supporting spontaneous thought generation in both dreaming and free association. Synchronization among the medial prefrontal cortex, precuneus, and posterior cingulate cortex provides the scaffolding for internally directed mental content, independent of external sensory input [7,18,41]. This network exhibits characteristic hyperconnectivity during REM sleep, facilitating spontaneous thought processes similar to mind-wandering observed during wakefulness [18,25].

Second, prefrontal dissociation represents a defining feature of both states. Systematic reduction in DLPFC activity during REM sleep constitutes one of the most robust neuroimaging findings [9,20,22]. The DLPFC supports executive functions, including working memory and cognitive control; its deactivation during REM reduces top-down constraint on posterior cortical activity, allowing more automatic, associatively driven processes to predominate. Analogous patterns occur during free association in waking states, where unconstrained word production shows decreased executive control while DMN and semantic processing areas increase [28,29,30].

Third, the amygdalo-hippocampal complex modulates the emotional and mnemonic content of unconscious processing. These structures shape the qualitative character of emerging mental content through their interaction with cortical networks [23,42,43]. The relative balance among these three components determines the specific phenomenology of different unconscious states.

Mechanisms and Dynamics

The functional microstates identified during REM sleep, alternating between tonic and phasic periods, suggest that unconscious processing exhibits systematic temporal dynamics [24]. Tonic REM periods maintain some residual alertness capacity and sensory processing, while phasic REM periods show intensified internal processing with greater isolation from external input [24].

Structural and functional studies reveal that greater mean diffusivity of the left amygdala correlates with shorter dream reports and lower emotional load, suggesting that microstructural integrity in this region directly influences access to emotional sources during dreaming [23]. More intriguing is the hemispheric dissociation: left amygdala volume correlates with narrative elaboration, while right amygdala measures associate with content bizarreness [23]. This lateralization suggests a functional division where left hemisphere structures support linguistic-narrative organization of dream content, while right hemisphere components contribute to spatial, emotional, and non-linear aspects.

This pattern raises questions that are not addressed in the current literature. If the left DLPFC shows reduced activity during REM while the left amygdala processes narrative elements, what coordinates this apparent contradiction? One possibility involves subcortical structures, particularly the thalamus, serving as relay stations that maintain functional coordination even when cortical control is diminished. Thalamo-cortical connectivity modulates the generation of both imagery and narrative content by activating visual and auditory networks [24].

Semantic networks operate through spreading activation in both dreaming and free association, accounting for the emergence of unexpected associations [27,28,31]. The anterior temporal lobe and angular gyrus serve as hubs in this process, integrating semantic information across modalities [27,28]. When prefrontal constraint diminishes, activation can spread more freely through these networks, following associative paths that would normally be inhibited during goal-directed thought.

Theoretical Frameworks and Compatibility

The neural substrates identified here bear interesting relationships to earlier psychological theories emphasizing dynamic unconscious processes, though these relationships require careful examination. Freud's topographic model positioned the unconscious as primarily a repository of repressed material, with dreams serving as disguised expressions of unacceptable wishes [1-3]. The neurobiological findings reveal a more complex picture. While reduced prefrontal control does allow expression of content that might be inhibited during wakefulness, there is no evidence for a specialized repression mechanism. Rather, the data suggest that decreased executive constraint simply permits the intrinsic dynamics of semantic and emotional networks to operate more freely [28-31].

Luis Cencillo's conceptualization of "unconscious life" as an active system of affective-cognitive processing shows interesting parallels with observed patterns of neural activity [4-6]. His emphasis on personalized "codes for interpreting reality," psychological and cultural structures that each individual develops to make sense of the world and their experience, resonates with findings of substantial inter-individual variability in DMN connectivity patterns [7]. His description of affective primacy finds potential correlates in amygdalo-hippocampal hyperactivation during REM sleep [9]. Yet these apparent convergences must be interpreted cautiously. Cencillo developed his framework through clinical observation, not from neurobiological predictions. The correspondence might reflect successful clinical intuitions about mental architecture, or it might demonstrate the flexibility of psychoanalytic concepts in being mapped onto diverse empirical findings.

What remains clear across theoretical frameworks is that substantial mental activity continues outside conscious awareness and executive control. However, a neural network activation spreading across milliseconds differs qualitatively from the psychic elaboration that unfolds in therapeutic sessions over months or years [44]. This difference in scale suggests that neurobiological and psychoanalytic descriptions address different levels of analysis, which may not map onto each other in straightforward ways.

Methodological Limitations

The limitations identified in this review reflect fundamental challenges inherent to neuroscientific investigation of subjective states. Dependence on self-reports for dream content inevitably introduces a temporal delay between the experience and its verbal description, during which secondary elaboration and confabulation can reshape the original experience [45,46]. Alternative approaches exist, including serial awakening protocols, but each carries methodological complications, and none entirely solves the problem of accessing subjective content that occurred in the absence of reportable consciousness.

The problem of inferring mental processes from patterns of brain activation presents another fundamental challenge. Functional neuroimaging reveals correlations between neural activity and mental states, but correlation does not specify the direction of causation, nor does it reveal the computational operations being performed by activated regions. This problem of "reverse inference" has been extensively documented, yet many studies continue to make such inferences without adequate justification [47].

Each neuroimaging technique brings specific limitations. Functional MRI offers spatial resolution sufficient to distinguish cortical regions but operates at temporal scales far slower than neural processing. EEG provides superior temporal resolution but poor spatial localization. Intracranial recordings offer both temporal and spatial precision but sample only a limited number of regions where electrodes are placed, typically in clinical populations with epilepsy, introducing potential sampling bias [48].

Methodological heterogeneity across studies further complicates the synthesis of findings. Differences in acquisition parameters, preprocessing pipelines, statistical thresholds, and analysis strategies can substantially affect results. The ecological validity of laboratory-based dream studies deserves particular attention. Neuroimaging environments impose multiple constraints (physical restrictions, scanner noise, artificial sleep schedules, and unfamiliar surroundings) that likely affect both sleep architecture and dream content [49].

Clinical Implications

The neural mechanisms identified here have potential relevance for several clinical conditions. Patients with post-traumatic stress disorder (PTSD) frequently experience nightmares characterized by repetitive, emotionally intense content. Neuroimaging studies in PTSD demonstrate altered patterns of amygdala-prefrontal connectivity, with reduced top-down regulation of limbic responses [50]. Whether the specific patterns observed during normal dreaming differ systematically from those in PTSD nightmares could inform our understanding of nightmare pathophysiology.

Imagery rehearsal therapy for nightmares might operate by strengthening prefrontal-limbic connections that provide greater executive control over emotional content during sleep. This hypothesis could be tested through pre- and post-treatment neuroimaging, examining whether successful therapy correlates with changes in these connectivity patterns [51].

Major depressive disorder involves well-documented alterations in sleep architecture, including shortened REM latency and increased REM density [50]. Some theories propose that depressive rumination shares neural mechanisms with both dreaming and mind-wandering, involving excessive DMN activity with insufficient regulation by attentional control networks [50]. If this model holds, interventions targeting DMN connectivity, through neurofeedback, transcranial magnetic stimulation, or pharmacological agents, might affect both waking rumination and dream content.

Ethical considerations deserve explicit attention. Interventions targeting unconscious processes raise questions about autonomy, consent, and personal identity. If certain patterns of unconscious activity contribute to a person's characteristic ways of thinking and feeling, interventions that alter these patterns might change aspects of personal identity. The history of psychoanalysis includes multiple examples of therapeutic overreach, where clinicians' theoretical commitments led to harmful interventions. As neuroscience provides new tools for intervening in mental processes, maintaining appropriate humility about our understanding and respecting patient autonomy become critical.

From a clinical neurosurgical perspective, patients undergoing evaluation for epilepsy surgery with intracranial electrode placement represent a unique opportunity. These individuals require monitoring over multiple days, during which both waking and sleeping states can be recorded with unprecedented spatial and temporal precision [52]. Such work could provide the most direct evidence about shared neural mechanisms between these states.

Future Directions

The relative lack of studies directly comparing dreaming and free association in the same individuals represents a significant gap. Experimental designs that evaluate both phenomena in the same participants could determine whether individuals who show more remote semantic associations during free association also report dreams with more bizarre content, or whether high DMN connectivity predicts both phenomena.

Pre-sleep manipulation studies offer another promising avenue. If semantic networks primed before sleep influence subsequent dream content, this would provide strong evidence for shared mechanisms with free association. Modern computational linguistics and natural language processing techniques could quantify the semantic distance between pre-sleep priming material and subsequent dream reports with greater precision than previous methods allowed [53].

The development of improved methods for objective measurement of dream content represents a crucial technical challenge. Machine learning techniques have begun to decode visual dream content from brain signals during sleep [19,35], but these methods currently work only for simple visual elements and require extensive individual calibration. Extending such approaches to more complex dream features would require substantial methodological advances but could eventually reduce reliance on subjective reports.

Computational modeling offers possibilities for testing mechanistic hypotheses about content generation during unconscious states. Neural network models that generate text or images through processes analogous to spreading activation in semantic networks could help validate proposed mechanisms [54-56]. Pharmacological studies could help dissociate components of the neural systems involved. Medications that modulate specific neurotransmitter systems produce characteristic changes in sleep architecture and dream content. Systematic investigation of how such manipulations affect both dreaming and free association could reveal the neurochemical basis of their shared mechanisms.

Beyond these methodological advances, a fundamental question concerns the generalizability of findings derived predominantly from Western populations. The neural substrates identified throughout this review (DMN architecture, prefrontal-limbic dynamics, and thalamo-cortical connectivity) are evolutionarily conserved across human populations. Yet the cultural practices surrounding sleep, dream interpretation, and the value attributed to unconscious processes vary substantially across human societies. This raises an empirical question that current literature has largely neglected: whether sustained, culturally transmitted practices might modulate the functional organization of these circuits through experience-dependent plasticity. Addressing this question requires extending neuroimaging research beyond its current demographic boundaries, a challenge that carries implications not only for theoretical completeness but also for the clinical applicability of any interventions derived from these findings.

Cultural Dimensions: An Untested Hypothesis

Available evidence suggests that the neural substrates involved in the regulation of sleep and wakefulness exhibit remarkable evolutionary conservation. In this regard, Guan et al. (2006) demonstrated that sleep deprivation induces widespread transcriptional changes, particularly in genes associated with synaptic plasticity, neuronal excitability, and energy metabolism, thereby supporting the existence of universal molecular mechanisms in sleep homeostasis. Nevertheless, upon this shared biological foundation, dream phenomenology displays pronounced cultural variability. Cross-cultural studies have documented systematic differences in recall frequency, emotional tone, narrative coherence, and the significance attributed to dreams. For instance, populations with elaborated dream traditions, such as Australian Aboriginal communities, report higher recall frequency and more complex narrative integration compared to Western urban populations. This convergence between biological conservation and cultural diversity underscores the need for interdisciplinary approaches that integrate the neurobiology of sleep with the anthropology of dream experience [56-59].

Interpreting these findings requires caution. Nearly all cross-cultural dream research relies on self-report, which confounds potential neural differences with cultural practices of encoding, elaboration, and selective reporting. A culture that values dreams and possesses a rich descriptive vocabulary may report more detailed dreams not because sleep neurobiology differs, but through enhanced attention, memory, and articulation. Distinguishing neural from reporting differences requires neuroimaging studies with standardized protocols across cultures, work conspicuously absent from current literature.

Cultural niche construction theory proposes that populations actively modify their environments, including symbolic ones, subsequently shaping their own development [60,61]. Applied to dreaming, this framework predicts that cultural practices around sleep, dream interpretation, and emotional regulation could influence developmental calibration of relevant neural systems. Meditation practitioners show altered DMN connectivity, demonstrating that sustained culturally transmitted practices can reshape functional architecture [62,63].

Several neurobiologically plausible mechanisms could mediate such influences through activity-dependent plasticity across individual lifespans [62,63]. However, the current evidence compels honesty: cultural modulation is neurobiologically plausible and generates testable predictions but lacks direct empirical validation. The reviewed literature includes no neuroimaging studies systematically comparing sleep architecture across culturally distinct populations while controlling for socioeconomic status, education, sleep quality, or psychiatric comorbidity. Until such research is conducted, claims about cultural modulation of dream neurobiology remain theoretically motivated hypotheses rather than empirically established conclusions.

## Conclusions

This review documents clear convergences in neural substrates supporting dream generation and free associative thought: both engage DMN structures, show limbic hyperactivation with prefrontal deactivation, and involve spreading activation through semantic networks with reduced executive constraint. These empirical regularities provide secure foundations for continued investigation. However, translating neuroscientific observations into broader psychological or cultural theories requires methodological caution. Neural activation patterns represent necessary substrates but do not fully explain subjective content or meaning. When connecting contemporary findings with earlier psychological theories, we propose interpretive hypotheses rather than demonstrate empirical validations. The relationship between levels of analysis presents particular challenges. A pattern of neural activation spreading across milliseconds operates at temporal scales vastly different from the psychic elaborations that unfold in clinical settings over months. A distributed network showing functional connectivity in fMRI differs fundamentally from a psychological construct such as "the unconscious." Some correspondence may exist between these levels, but the mapping is not straightforward.

Future progress requires experimental designs specifically aimed at testing integrative hypotheses rather than simply noting apparent convergences. The gap between demonstrating neural correlates and explaining subjective experience, the "hard problem" of consciousness, remains as challenging as ever. We can identify brain regions active during dreaming, measure their connectivity patterns, and even decode some aspects of dream content from neural signals. Yet why these particular patterns of neural activity give rise to the subjective feel of dreaming remains mysterious. This review characterizes the neural substrates involved, which represent a necessary first step, even if insufficient for a complete explanation. The clinical implications of these findings merit careful development. The identification of neural markers for unconscious processing suggests possibilities for novel interventions, but translation from basic science to therapeutic application requires additional validation and careful attention to ethical concerns. Future advances will depend on more direct experimental integration of dream and free association paradigms, improved methods for objective measurement of subjective content, computational models that generate testable predictions, and cross-cultural studies with adequate methodological rigor.
